# Exercise enhances memory consolidation in the aging brain

**DOI:** 10.3389/fnagi.2014.00003

**Published:** 2014-02-03

**Authors:** Shikha Snigdha, Christina de Rivera, Norton W. Milgram, Carl W. Cotman

**Affiliations:** ^1^Institute for Memory Impairments and Neurological Disorders, University of CaliforniaIrvine, CA, USA; ^2^CanCog Technologies Inc., Toronto, ONCanada

**Keywords:** exercise, memory consolidation, aging, concurrent discrimination, object location, novel object recognition, reversal learning, dogs

## Abstract

Exercise has been shown to reduce age-related losses in cognitive function including learning and memory, but the mechanisms underlying this effect remain poorly understood. Memory formation occurs in stages that include an initial acquisition phase, an intermediate labile phase, and then a process of consolidation which leads to long-term memory formation. An effective way to examine the mechanism by which exercise improves memory is to introduce the intervention (exercise), post-acquisition, making it possible to selectively examine memory storage and consolidation. Accordingly we evaluated the effects of post-trial exercise (10 min on a treadmill) on memory consolidation in aged canines both right after, an hour after, and 24 h after acute exercise training in concurrent discrimination, object location memory (OLM), and novel object recognition tasks. Our study shows that post-trial exercise facilitates memory function by improving memory consolidation in aged animals in a time-dependent manner. The improvements were significant at 24 h post-exercise and not right after or 1 h after exercise. Aged animals were also tested following chronic exercise (10 min/day for 14 consecutive days) on OLM or till criterion were reached (for reversal learning task). We found improvements from a chronic exercise design in both the object location and reversal learning tasks. Our studies suggest that mechanisms to improve overall consolidation and cognitive function remain accessible even with progressing age and can be re-engaged by both acute and chronic exercise.

## INTRODUCTION

Observational studies have identified physical activity (exercise) as one of the three main modifiable risk factors for developing AD and dementia (Barnes and Yaffe, 2011). Exercise is known to promote brain health and delay the onset of cognitive decline in aging and AD. Exercise has been reported to increase synaptic plasticity and long-term potentiation (LTP; van Praag et al., 1999; Liu et al., 2011; Dao et al., 2013), upregulate brain derived neurotrophic factor (BDNF) expression, and reduce accumulation of reactive oxygen species (Radak et al., 2001; Berchtold et al., 2002). Thus, exercise primes the brain to access mechanisms that can improve cognitive faculties and reduces the detrimental effects of molecular and synaptic changes that occurs with age (van Praag et al., 2005; Kronenberg et al., 2006; Cotman and Berchtold, 2007; Cotman et al., 2007; Kannangara et al., 2011).

Using a canine model of aging, we have shown that behavioral enrichment consisting of exercise, enriched housing and cognitive enrichment can significantly improve the cognitive capacity of old beagles. In these past studies we demonstrated an association between long-term enrichment and exercise and improved cognitive function (Milgram et al., 2004; Snigdha et al., 2011; Fahnestock et al., 2012). Our results were indicative of a causal link between exercise and improved learning and memory.

The present experiments sought to explore this relationship further to better understand how exercise enhances memory. One broadly accepted model of memory formation assumes that memory occurs in phases which include, an acquisition phase (learning), followed by an intermediate labile phase, and then a process of consolidation which leads to long-term memory formation (McGaugh, 1966, 2000; Bekinschtein et al., 2007; McKenzie and Eichenbaum, 2011). Memory consolidation occurs by a cascade of molecular and cellular events which results in stable synaptic and network modifications (Davis and Squire, 1984; McGaugh, 2000; Bekinschtein et al., 2007; Park and Poo, 2013). In these experiments our main focus was on evaluating the role of exercise in promoting memory consolidation. While the effect of exercise on cognitive function has been extensively studied, the specific role of exercise in promoting consolidation is yet to be explored.

One effective way to examine memory consolidation is the use of post-trial interventions (Cahill and Alkire, 2003; Segal et al., 2012). By introducing exercise after the learning phase, it is possible to test effects of exercise on memory storage and consolidation selectively in such intervention studies. Therefore, we designed a study to examine the effects of an acute post-learning exercise intervention in aged animals. It has been suggested that consolidation requires a significant involvement of the hippocampus in the first stages and later requires a greater involvement of the task-relevant cortical areas (McKenzie and Eichenbaum, 2011). Thus, due to the task specificity and time-contingency, it is critical to use different behavioral tasks to assess the effects of an intervention on consolidation. Consequently, in this study we evaluated the effects of exercise on memory consolidation both right after and 24 h after the exercise training in a several different behavioral paradigms.

Specifically, we evaluated the effects of acute exercise in a concurrent discrimination task which is known to be medial temporal lobe (MTL)-dependent in humans (Squire et al., 1988; Hood et al., 1999). MTL involvement in memory processes has been studies for more than half a century now, particularly after the case of patient HM was reported (Scoville and Milner, 1957). The networks of MTL structures include the hippocampus and surrounding cortices such as perirhinal, entorhinal, and parahippocampal cortices (Squire et al., 2004). Many of these regions, including the hippocampus (Bach et al., 1999; Lister and Barnes, 2009) and perirhinal cortex (Burke et al., 2011, 2012) are known to be susceptible to aging. In this study we utilized tasks which would map to these specific regions of the MTL. Specifically, the object location memory (OLM) and novel object recognition (NOR) tasks which rely on the hippocampus (Holdstock et al., 2002; Mumby et al., 2002a) and perirhinal cortex respectively (Mumby et al., 2002b; Winters and Bussey, 2005) were used to test and confirm the effects of exercise on memory consolidation. While the OLM task has been shown to be sensitive to age in mice and humans, there have been no studies documenting age-related effects in NOR. Furthermore, neither OLM nor the NOR task has been demonstrated in dogs previously and were specifically developed for this study. A major advantage of using the OLM and NOR tasks is that they allow for testing the effects of single bouts of exercise and do not require pre-training. This provides an excellent tool to study consolidation processes. We also conducted a 2 week exercise study to compare the effects of acute vs. longer term exercise on OLM.

It is now widely accepted that the network of MTL structure are implicated in many but not all kinds, of cognitive and memory functions (Squire, 2004). Thus, in order to test whether the effect of exercise impacted regions other than the hippocampus, aged animals were tested on a reversal learning task. The reversal learning task is a frontal lobe-dependent task (McAlonan and Brown, 2003; Stalnaker et al., 2007) and has been previously described in dogs in several communications (Milgram et al., 1994; Tapp et al., 2003; Head et al., 2008). Performance on this task predominantly relies on executive functions (Lai et al., 1995) and assess loss of inhibitory control in aging (Tapp et al., 2003; Snigdha et al., 2013). It has also been suggested that inhibitory failure disrupts several aspects of overall cognitive function (e.g., working memory) and interferes with encoding and retrieval processes (Hasher and Zachs, 1988; Buckner, 2003). Hence, we evaluated the effect of exercise on the reversal learning task to assess if an intervention that impacts memory consolidation also improves performance of aged animals in a task which relies on inhibitory control.

## MATERIALS AND METHODS

### SUBJECTS

Twenty-two beagles ranging in age at the start of the study from 10.1 to 11.1 years (mean = 10.6 years, SD = 0.23, eight males/14 females) were obtained from the colony at CanCog Technologies Inc. (Toronto, Canada) and housed at the Cancog site during the study. The study protocol was approved by the CanCog Technologies Institutional Animal Care Committee, and it followed the guidelines of the Ontario Ministry of Agriculture. All animals used for the study were visually examined daily by trained veterinary animal personnel and research staff. All dogs had similar cognitive testing experience, which included pre-training as described previously (Milgram et al., 1994). Veterinary examinations were conducted on all dogs prior to the start of the study to assess their general health and to ensure that visual, auditory, and motor functioning were not compromised. All animals were group-housed indoors, had free access to water, and were fed once daily. The dogs’ dietary history included three different diets: standard adult maintenance diet (Purina Pro Plan® Chicken and Rice), the standard diet supplemented with mitochondrial co-factor and antioxidant supplements, or the standard diet supplemented with lipoic acid. In the current study, the two treatment groups (exercise, control) both contained animals from all three dietary conditions and animals from all three dietary groups were distributed equally between control and exercise groups for this study. Housing temperature was held at 21 ± 6°C and relative humidity levels ranged between 15 and 75%.

### GROUP ASSIGNMENTS

All animal in the study were trained to run on the treadmill, following which, animals were divided into two groups: exercise (*n* = 11) and control (non-exercised; *n* = 11). For the exercise group, dogs underwent one daily session of treadmill exercise (10 min) as described below, while control dogs were placed in a metabolic or veri-kennel for 10 min. Both the treatment groups contained a balanced group of animals from all three dietary conditions.

### EXERCISE INTERVENTIONS TESTED

This study investigated the efficacy of different exercise intervention strategies in improving cognitive performance in aged dogs. First, we assessed the effects of post-training exercise session (10 min) on subsequent consolidation of the cognitive task. Subjects were tested at 2 timepoints: immediately after the exercise session (concurrent discrimination task), or 1 h after the exercise session (OLM and NOR tasks), and 24 h after the exercise session. In addition to testing how post-learning acute exercise affects cognitive performance, we investigated the effects of chronic daily exercise on cognitive function using two different behavioral approaches. It should be noted that colony animals get about 30 min of exercise daily (i.e., they are let out of the pens or metabolics to run freely in the aisles) and prior to starting on the exercise study all dogs were given this 30 min of daily exercise. Furthermore, all dogs in this study have previous cognitive testing experience and have been able to perform normally on many cognitive tasks. We have also found that cognitive testing itself is a very positive experience for the dogs and it was evident from daily observations of the entire colony that the dogs were neither depressed nor deprived at the start of this study.

### EXERCISE Protocol

#### Habituation

Exercise group animals were first acclimated to the treadmill for 5 days prior to study initiation to allow animals to build endurance and to decrease acute exercise related stress. The habituation began with 4 min of exercise (including 1 min of warm up and 1 min to cool down) and was increased 2 min/day to eventually total 10 min. Animals were equipped with a harness prior to exercise and were allowed to climb on the treadmill voluntarily, or were lifted onto treadmill if assistance was needed. A leash was attached to the harness so that the animal’s hind legs did not leave the end of the treadmill belt while walking/trotting.

#### Daily exercise session

Animals were allowed to first warm up for 1 min at the ideal walking pace for that animal, and then exercised at a fast walk or trotting pace for 8 min. The speed was dependent on the animal’s abilities. A 1 min cool down was provided at a speed equal to or lower than the warm up speed, depending on the animal’s abilities. The total exercise time was 10 min.

#### Cognitive tasks

Cognitive function was tested using four cognitive tasks: concurrent testing, reversal learning, OLM, and NOR, in combination with acute or chronic exercise. Effects of acute post-learning exercise on consolidation were tested using the concurrent discrimination task, OLM and NOR, and chronic exercise effects were tested using ORM and Reversal learning task.

#### The order of testing was as follows

concurrent discrimination (acute exercise, *n* = 11/group), reversal learning (chronic exercise, *n* = 11/group), OLM (acute exercise, *n* = 6 for control group and 5 for exercise group), NOR (acute exercise, *n* = 8/group at 1 h, *n* = 7 for control and 8 for exercise groups at 24 h), OLM (chronic exercise, *n* = 6 for control and 8 for exercise groups at 1 h, *n* = 6/group at 24 h). The testing phases were spaced temporally such that at least 4–6 weeks separated each exercise/cognitive phase from the preceding test phase. The testing took place over a 2 year period, and because dogs were old, there was some group attrition due to vision loss, arthritis, cancer, death, or other factors that rendered the animal incapable of participating (e.g., animals that did not engage in the task were also excluded from the analysis). Sample sizes for each testing parameter are indicated in the figure legends.

### TESTING ARENAS

Cognitive testing for the *concurrent discrimination and reversal learning tasks* was conducted in a canine adaptation of the Wisconsin general testing apparatus, as described previously (Milgram et al., 1994). Briefly, the apparatus consisted of a large holding area where the dog was housed during testing, which was separated from the experimenter by a wooden screen containing a one-way mirror and a hinged door at the bottom. A Plexiglas stimulus tray containing three food wells was pushed through the hinged door by the experimenter so that the dog could access the stimuli and food rewards by sticking its head through adjustable stainless steel gates at the front of the holding area. The tray was removed out of sight during delay and inter-trial intervals. Food reward for correct responses during cognitive testing consisted of approximately 1 g of wet dog food (Hill’s P/D). To mask the presence of the food reward in the negative food wells, the undersides of all stimuli were baited with the same food such that, while able to smell it, the animals could not see or eat it. For all tasks, a partial correction procedure was used in which the dogs were permitted to correct their response after making an error once each session. *OLM and NOR testing* took place in an open field room (approximately 8 × 9 ft) equipped with two cameras to record movement for data analysis.

### CONCURRENT DISCRIMINATION TASK

The concurrent discrimination task measures the ability to remember object–object associations in the presence of active interference. Animals were assessed for object preference prior to discrimination testing, as described below.

#### Preference testing

Prior to cognitive testing, animals were presented with a pair of objects, and tested for their preferred object of the pair. Animals were given 20 presentations of four different object pairs, in which each of the object pairs were presented on five occasions. Responses to each object were recorded and all responses were rewarded with a food reward. For each object pair, the object selected most frequently was designated the preferred object and the object selected less frequently was designated the non-preferred object.

#### Concurrent discrimination testing

One session consisted of 24 trials, in which the animal was presented with an object pair and was rewarded for choosing their non-preferred object. The 24 trials were separated by a 15 s inter-trial interval, and consisted of four different object pairs presented sequentially six times. Each object pair differed in three dimensions: color (gray or white), size (small, medium, large), and shape. On a first trial, of each object pair, a correction procedure was used, with subjects being allowed to correct their response if they chose incorrectly. Subjects received a maximum of 30 s to respond to each trial. If a response was not made in the first 15 s of a trial, verbal coaxing was initiated. A non-response was recorded if the animal did not respond within 30 s.

#### Testing procedure with exercise

**FIGURE 1:**
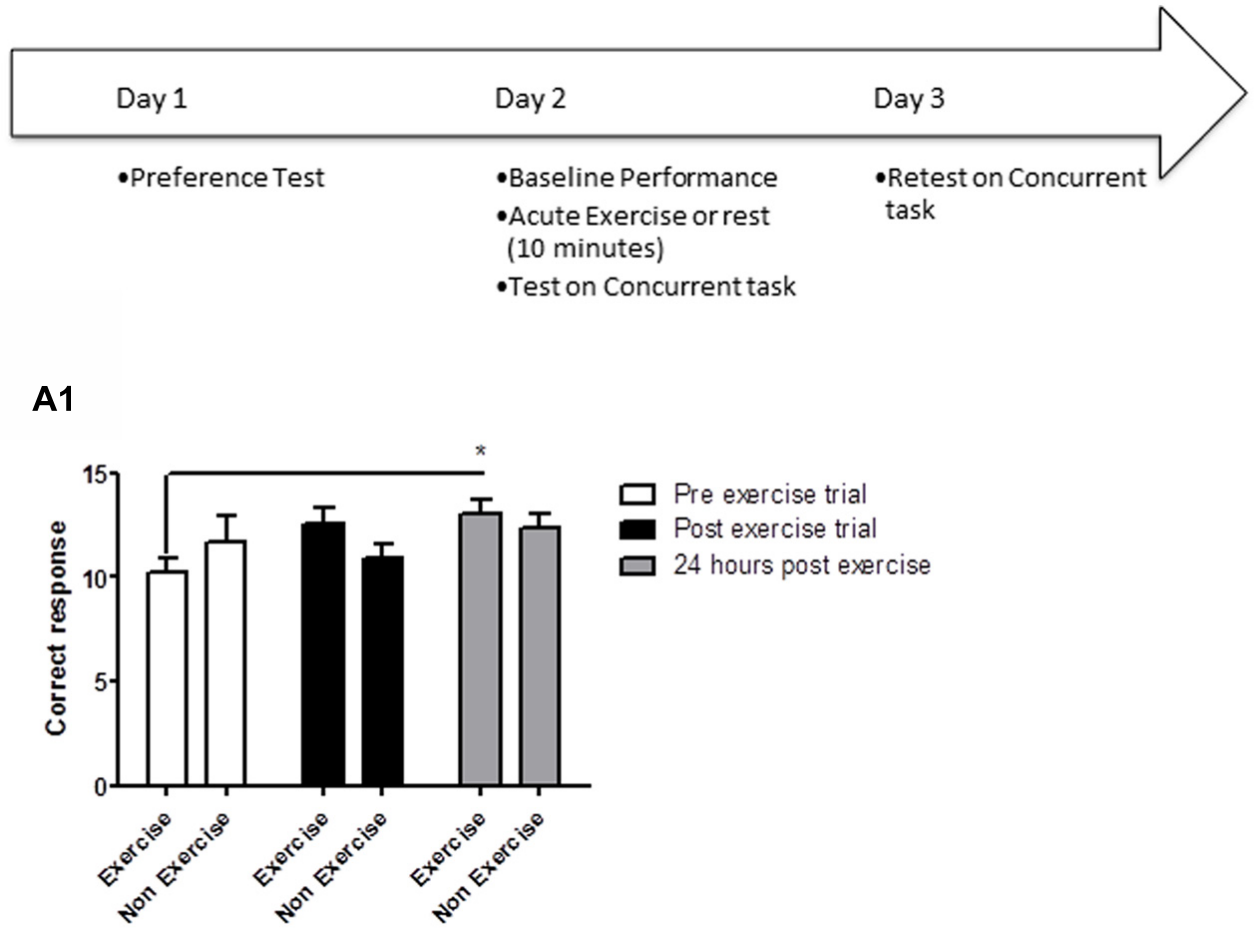
**Schematic showing testing on the concurrent discrimination task after acute exercise.** Animals were tested on the concurrent task over the course of 3 days. Day 1: preference testing, Day 2: baseline performance, followed by 10 min exercise or rest (control group), and retesting in the concurrent discrimination task immediately after the 10 min exercise/rest interval. Day 3: re-testing 24 h after the preceding day’s exercise session. **(A1)** Effect of 10 min of post-trial exercise on performance of aged beagles in the concurrent discrimination task. The figure shows the number of correct response in the concurrent discrimination task conducted first prior to exercise, then immediately after exercise, and finally at 24 h after the exercise training. At 24 h post-exercise the animals in the exercise group were significantly improved compared to the pre exercise trial. *p < 0.05. Significant difference from pre exercise trial. *n* = 11/group, Error bars show ± SEM.

Animals were tested over the course of 3 days. *Day 1:* preference testing, *Day 2:* baseline performance, followed by 10 min exercise or rest (control group), and retesting in the concurrent discrimination task immediately after the 10 min exercise/rest interval. *Day 3:* re-testing 24 h after the preceding day’s exercise session (**Figure 1**).

### OBJECT LOCATION MEMORY

This task relies on spontaneous exploratory activity and the inherent preference for investigating novelty. Memory is evaluated by the differences in the exploration time of identical objects that are in novel or familiar locations. The effect of exercise on memory consolidation was tested at 1 and 24 h after an exercise exposure.

**FIGURE 2:**
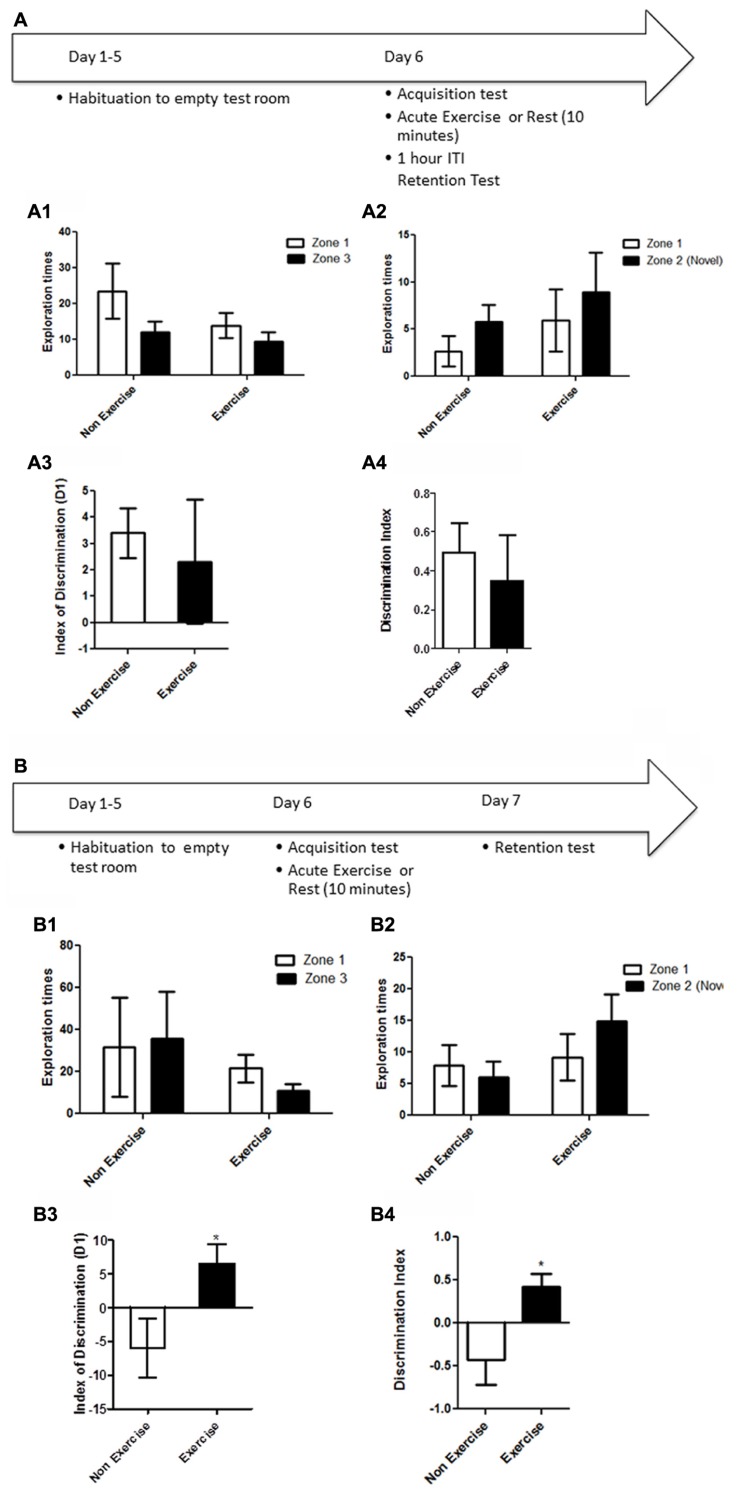
**Schematic showing testing on the OLM task after acute exercise.** Animals were first habituated over 5 days. Following habituation, animals were given a 5 min of acquisition session to explore the testing room with two identical objects placed in the room. Immediately following the acquisition session, animals in the exercise group were given 10 min treadmill exercise, while control animals remained sedentary. One hour later, memory was assessed. **(B)** In a separate experiment, memory was assessed 24 h after the exercise/rest session: animals underwent an acquisition session (5 min), followed by exercise/rest (10 min), then 24 h later a memory test where the location of one of the objects had been changed. **(A)** Effect of post-trial exercise on performance of aged beagles 1 h after exercise in the object location memory task. **(A1)** Object exploration during the acquisition phase of the object location memory task. There was no difference in object exploration of the two objects in the acquisition phase. **(A2)** One hour after the 10 min exercise regimen neither the exercise group nor the control group could successfully discriminate between the novel and familiar location. **(A3)** The index of discrimination also did not show any significant difference between the two group, 1 h after the exercise. **(A4)** Discrimination index also did not show any significant difference between the two group, 1 h after the exercise. *n* = 5–6/group. Error bars show ± SEM. **(B)** Effect of post-trial exercise on performance of aged beagles 24 h after exercise in the object location memory task. **(B1)** Object exploration during the acquisition phase of the object location memory task. There was no difference in object exploration of the two objects in the acquisition phase. **(B2)** Twenty-four hour after the 10 min exercise regimen the exercise group showed a trend for greater exploration of the novel location compared to control group, although the effect was not significant. **(B3)** The index of discrimination showed a significant difference between the two groups 24 h after exercise. **(B4)** Discrimination index showed a significant difference between the two groups 24 h after the exercise.*p < 0.05. Significant difference between exercise and control group, with only the animals in the exercise group successfully discriminating novel location from familiar. *n* = 6–8/group. Error bars show ± SEM.

Animals were first habituated over 5 days (10 min/day) to the empty open field testing room. Following habituation, animals were given a 5 min acquisition session to explore the testing room after two identical objects had been placed in diagonally opposite corners, and the time spent exploring each object was recorded. Immediately following the acquisition session, animals in the exercise group were given 10 min treadmill exercise, while control animals remained sedentary. One hour later, memory was assessed. Each animal was led back to the open field room where the location of one of the objects had been changed, and animals were allowed to explore the objects and test room for 5 min. The time spent exploring each object was recorded (**Figure 2A**).

In a separate experiment, memory was assessed 24 h after the exercise/rest session: animals underwent an acquisition session (5 min), followed by exercise/rest (10 min), and 24 h later a memory test in which the location of one of the objects had been changed (**Figure 2B**). In all experiments, the room and objects were cleaned before and after the acquisition and memory testing for each animal.

### NOVEL OBJECT RECOGNITION MEMORY

**FIGURE 3:**
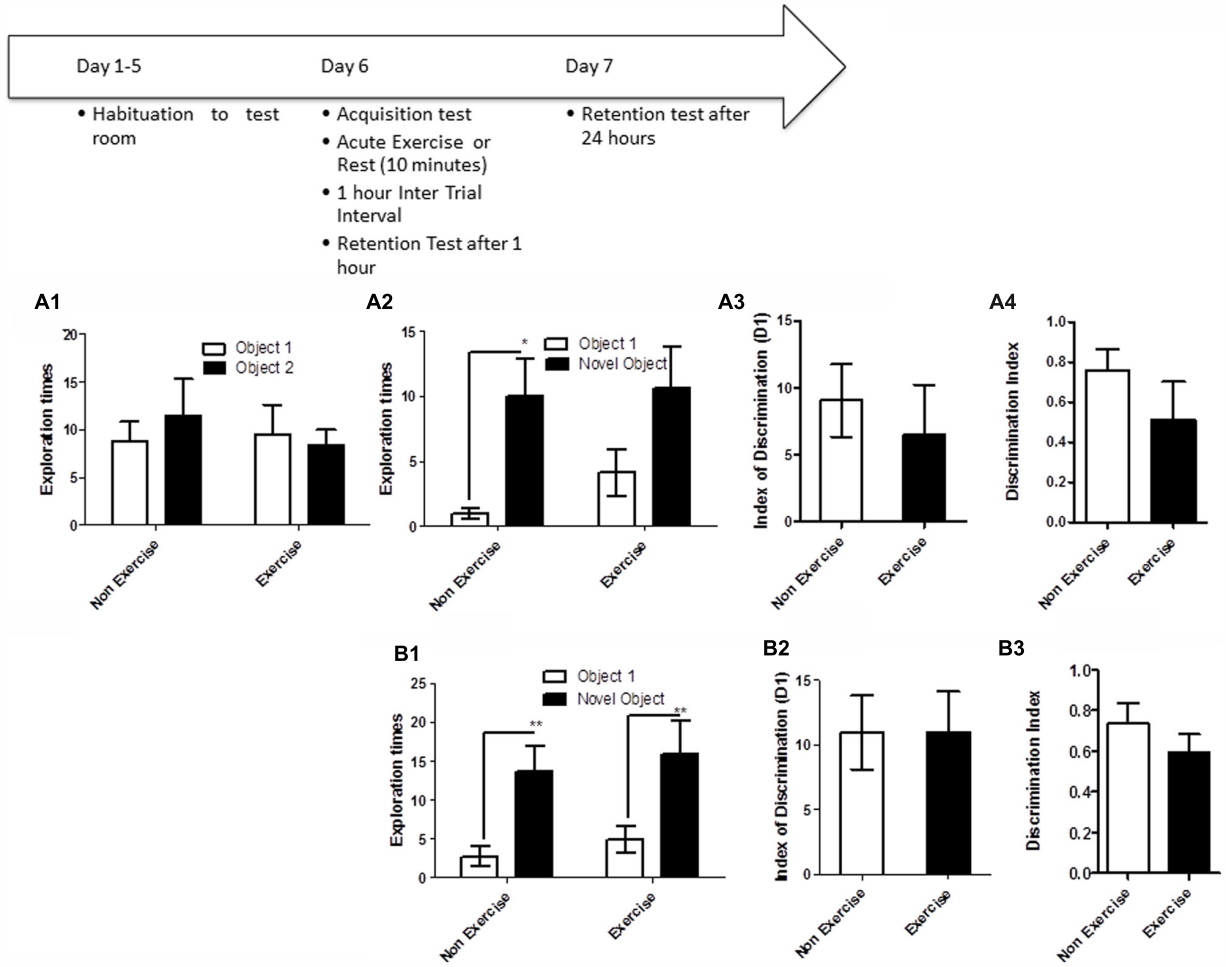
**Schematic showing testing on the NOR task.** Animals were first individually familiarized (10 min) with the empty open field test room. Following habituation, animals were given a 5 min acquisition session to explore the testing room after two identical objects had been placed in diagonally opposite corners, and the time spent exploring each object was recorded. Immediately following the acquisition session, animals in the exercise group were given 10 min treadmill exercise, while control animals remained sedentary. Memory was assessed 1 and 24 h after the exercise/rest session. **(A1–A3)** Effect of post-trial exercise on performance of aged beagles in the novel object recognition task. **(A1)** Object exploration during the acquisition phase of the novel object recognition task. There was no difference in object exploration of the two objects in the acquisition phase. **(A2)** One hour after the 10 min exercise regimen the control group could successfully discriminate between the novel and familiar objects, the exercise group also showed a similar trend albeit not quite significant. **(A3)** The index of discrimination did not show any significant difference between the two groups, 1 h after the exercise suggesting that both groups performed similarly on the task. **(A4)** Discrimination index also did not differ between the two groups, 1 h after the exercise. *p < 0.05. Significant increase in exploration of the novel object compared to familiar object in control animals. *n* = 8/group. Error bars show ± SEM. **(B1)** Twenty-four hour after the 10 min exercise regimen both the exercise and control groups showed significantly greater exploration of the novel object compared to familiar object. **p < 0.01. Significant difference between novel and familiar object exploration. **(B2)** The index of discrimination showed no significant difference between the two groups 24 h after exercise suggesting that both groups successfully discriminated the novel object from the familiar object. **(B3)** Discrimination index showed no significant difference between the two groups, 24 h after exercise. *n* = 7–8/group. Error bars show ± SEM.

Like the OLM task, this task relies on spontaneous exploratory activity and the inherent preference for investigating novelty. Memory is evaluated by the differences in the exploration time of novel and familiar objects. Animals were first individually familiarized (10 min) with the empty open field test room. Following habituation, animals were given a 5 min acquisition session to explore the testing room after two identical objects had been placed in diagonally opposite corners, and the time spent exploring each object was recorded. Immediately following the acquisition session, animals in the exercise group were given 10 min treadmill exercise, while control animals remained sedentary. Memory was assessed 1 and 24 h after the exercise/rest session (**Figure 3**). For memory assessment, each animal was led back to the open field room, where one of the objects had been replaced with a novel object, and animals were allowed to explore the objects and test room for 5 min. The time spend exploring each object was recorded. The room and objects were cleaned before and after the acquisition and memory testing for each animal.

### CHRONIC EXERCISE PRIOR TO LEARNING: OLM

**FIGURE 4:**
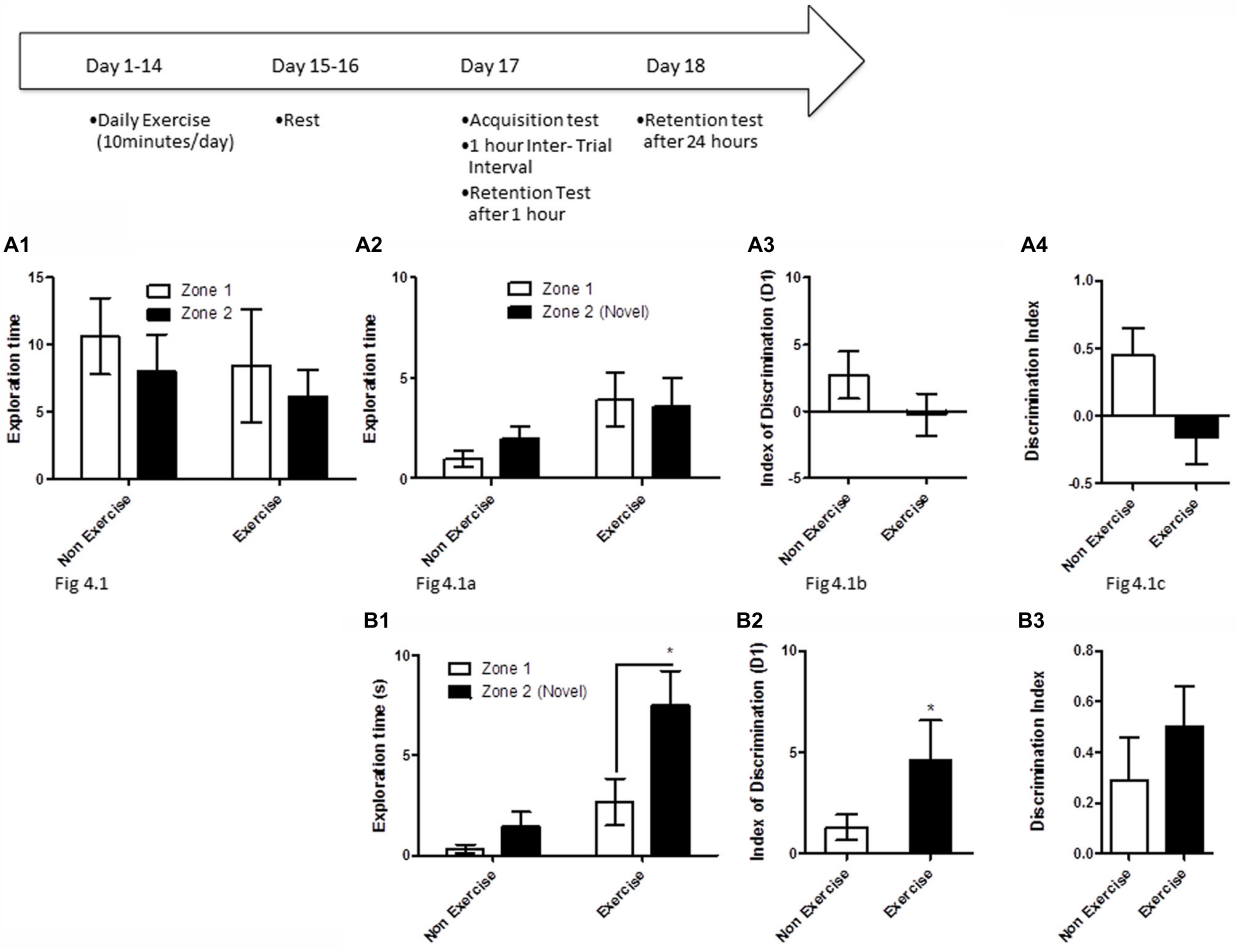
**Schematic showing testing on the OLM task after chronic exercise.** To test if chronic exercise improved hippocampal function, animals in the exercise group underwent daily treadmill exercise for 14 consecutive days. Two days later, all animals were given a 5 min acquisition session to explore two identical objects that had been placed in diagonally opposite corners of the testing room, and the time spent exploring each object was recorded. Retention memory was tested at 1 and 24 h after the acquisition trial. **(A2–B2)** Effect of 14 days of exercise on performance of aged beagles in the object location memory task conducted 48 h after last exercise session. **(A1)** Object exploration during the acquisition phase of the object location memory task. There was no difference in object exploration of the two objects in the acquisition phase. **(A2)** One hour after the acquisition animals were tested on the object location memory task and neither the exercise group nor the control group could successfully discriminate between the novel and familiar location. **(A3)** The index of discrimination also did not show any significant difference between the two group, 1 h after the exercise. **(A4)** Discrimination index also did not show any significant difference between the two groups, 1 h after exercise. *n* = 6–8/group. Error bars show ± SEM. **(B1)** Twenty-four hours after the acquisition phase or 48 h after the last exercise session, the exercise group showed greater exploration of the novel location compared to control group. **(B2)** The index of discrimination showed a significant difference between the two groups 24 h after acquisition. **(B3)** Discrimination index did not show any significant difference between the two groups, 24 h after acquisition. *p* < 0.05. *Significant difference between exercise and control group, with only the animals in the exercise group successfully discriminating between the novel and familiar locations. Error bars show ± SEM. *n* = 6/group.

To test if long-term exercise improves hippocampal function in aged dogs, animals in the exercise group underwent daily treadmill exercise for 14 consecutive days. Two days later, all animals were given a 5 min acquisition session to explore two identical objects that had been placed in diagonally opposite corners of the testing room, and the time spent exploring each object was recorded. Retention memory was assessed at 1 and 24 h after the acquisition trial (**Figure 4**). For memory assessment, one of the objects had been replaced with an object in a novel location as described above, and animals were allowed to explore the objects and test room for 5 min.

### CHRONIC EXERCISE CONCURRENT WITH LEARNING: REVERSAL TASK

**FIGURE 5:**
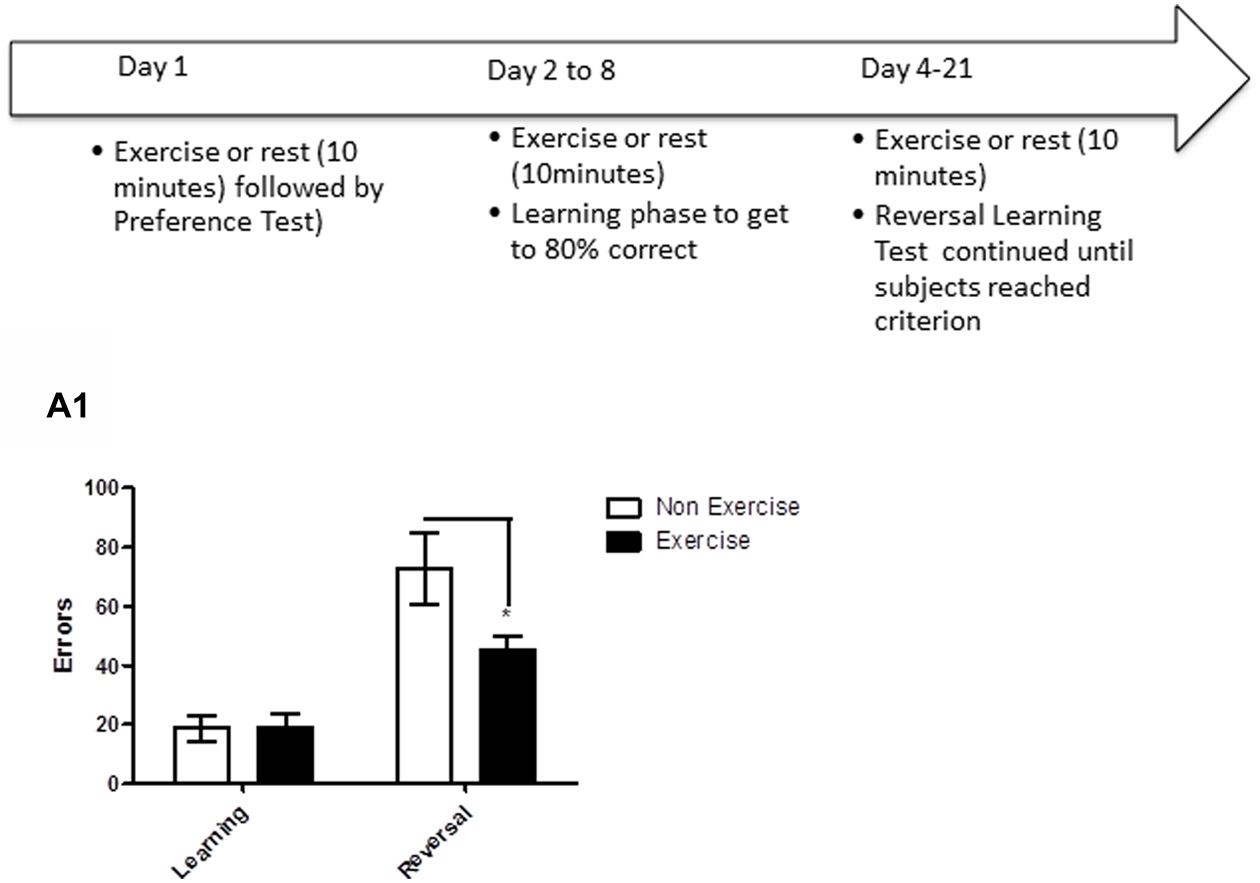
**Schematic showing testing of animals on the reversal learning task.** After identifying each animal’s preferred/non-preferred objects for a given object pair on day 1, animals learned to associate a positive food reward with the preferred object over the next week. Once the criterion of ≥80% correct responses was reached in a day’s session, reversal learning was initiated. Animals were tested on the reversal phase until the animal achieved reached criterion (≥80% correct in a single session). For animals in the exercise group, each dog underwent daily exercise (10 min treadmill) immediately prior to the test session, beginning the day of preference testing and continuing through reversal learning. Subjects in the control group remained sedentary in a metabolic or veri-kennel for 10 min. **(A1)** Effect of pre-trial exercise on performance of aged beagles in the reversal learning task. Performance on learning or discrimination phase of the task did not differ between control animals and those in the exercise group. There was a significant effect of task, in that, both groups made more errors in acquiring the reversal task compared to initial discrimination task. There was also a significant difference between the exercise and control group in the number of errors to reach criterion in the reversal phase of the task.*p < 0.05. Error bars show ± SEM. *n* = 11/group.

Reversal learning is frontal lobe-dependent task that requires the animal to disregard a previously learned object–object association, and to learn a new association. In this study, after identifying each animal’s preferred/non-preferred objects for a given object pair (as described above for the concurrent discrimination task), animals learned to associate a positive food reward with the preferred object. Each daily session consisted of 20 trials (15 s inter-trial interval) where subjects were presented with objects which differed in color and shape, but not in size. Errors were recorded, and once criterion was reached of ≥80% correct responses in a day’s session, reversal learning was initiated. During reversal learning, the non-preferred objects became the positive stimulus. Daily errors were recorded, and animals were tested on the reversal phase until the animal achieved reached criterion (≥80% correct in a single session). For animals in the exercise group, each dog underwent daily exercise (10 min treadmill) immediately prior to the test session, beginning the day of preference testing and continuing through reversal learning (**Figure 5**). Subjects in the control group remained sedentary in a metabolic or veri-kennel for 10 min. A correction procedure was allowed, such that on the first incorrect response of the first session of learning and reversal, subjects were allowed to correct their response. Days of exercise was determined by the number of days needed to reach criterion.

## STATISTICS AND ANALYSIS

All statistical analyses were conducted using GraphPad Prism. Repeated measures analysis of variance (ANOVA) or one-way ANOVA was used to assess the effects of exercise. Bonferroni *post hoc* test was conducted where necessary to examine group differences when a significant overall effect was found. Behavior marking was also scored manually while watching the animal in the room for confirmation. For concurrent discrimination and reversal learning tasks, the total number of correct responses and errors made to reach the criteria were used as the main measure respectively. For OLM and NOR tasks, the total time spent exploring objects were used as main measures. Index of discrimination (D1) was calculated by subtracting time spent exploring familiar object/location from time spent exploring novel object/location and discrimination index (DI) was calculated as the ratio of the difference between time spent exploring familiar object/location from time spent exploring novel object/location, and the sum of time spent exploring familiar object/location from time spent exploring novel object/location[(novel - familiar)/(novel + familiar)].

## RESULTS

### ACUTE POST-LEARNING EXERCISE: EFFECTS ON CONSOLIDATION

Three different tasks were used to assess the effectiveness of post-learning exercise in enhancing memory consolidation: concurrent discrimination learning, OLM and NOR.

### CONCURRENT DISCRIMINATION TASK

The concurrent discrimination task tested the ability of the aged dogs to learn a list of object-pair discriminations (24 trials: four object pairs presented six times). To evaluate if post-learning exercise improved performance, animals were tested on the concurrent task (baseline performance), underwent exercise (10 min), or rest (controls), and were retested both immediately after the exercise session and 24 h later.

Concurrent discrimination testing revealed that an acute session of post-learning exercise enhanced memory performance relative to control animals. A mixed factor ANOVA with treatment as the between subjects effect and time of testing as the within subjects factor revealed a significant effect of time [*F*_(2,40)_ = 3.05; *p* < 0.05], but not treatment [*F*_(1,20)_ = 0.16; *p* = NS]. The interaction between the two factors was not quite statistically significant [*F*_(2,40)_ = 2.5, *p* = 0.09]. *Post hoc* analysis demonstrating that exercise animals showed significantly better performance 24 h (*p* < 0.05) post-exercise, and a trend (*p* = 0.05) for better performance immediately after exercise (**Figure 1A1**). These data reveal that a single session of post-learning exercise improves memory performance, with a significant improvement observed 24 h after exercise. These data suggest that post-learning exercise facilitates consolidation of a MTL-dependent task.

### OBJECT LOCATION MEMORY

We next assessed if post-learning exercise improves performance of aged dogs in the OLM task. Testing consisted of an acquisition trial (5 min), immediately followed by exercise (10 min), or rest (controls), and assessment of performance effects at 1 h, or 24 h post-exercise intervention.

OLM memory testing revealed that an acute session of post-learning exercise improved memory performance relative to controls, but only after 24 h of consolidation. A mixed factor ANOVA with treatment as the between subjects effect and object location as the within subjects factor revealed no overall interaction [*F*_(1,8)_ = 0.43, *p* = NS] or main effect of treatment [*F*_(1,8)_ = 0.21, *p* = NS]. Although there was a significant effect of object location [*F*_(1,8)_ = 5.31, *p* < 0.05], *post hoc* analysis showed no difference between exploration of object locations for either the control or exercise groups (*p* = NS) in the acquisition phase of the task at 1 h (**Figure 2A3**). There was no significant interaction effect [*F*_(1,18)_ = 0.04, *p* = NS] or main effect of exercise [*F*_(1,18)_ = 1.16, *p* = NS] (**Figure 2A2**) or location [*F*_(1,18)_ = 0.95, *p* = NS] (**Figure 2A2**) at the 1 h post-exercise timepoint. There was also no significant difference in the index of discrimination between groups in the 1 h retention test (*p* = NS, **Figure 2A3**) or in the DI (*p* = NS, **Figure 2A4**).

For the retention test conducted 24 h after acquisition, there was no interaction [*F*_(1,12)_ = 3.15, *p* = NS; mixed factor ANOVA] or main effect of exercise [*F*_(1,12)_ = 0.73; *p* = NS, **Figure 2B1**] or location [*F*_(1,12)_ = 0.69; *p* = NS, **Figure 2B1**] in initial object exploration in the acquisition phases of the task, revealing that the two objects were equally attended to, and thus equally salient. A mixed factor ANOVA with treatment as the between subjects effect and object location as the within subjects factor revealed that the interaction effect between exercise and object location for the 24 h retention trial was not significant [*F*_(1,11)_ = 2.08; *p* = NS]. There was no significant effect of exercise [*F*_(1,11)_ = 2.35; *p* = 0.33, **Figure 2B2**] or object location [*F*_(1,11)_ = 0.54; *p* = NS] either. However, 24 h after acquisition, the index of discrimination (**Figure 2B3**) and the DI (**Figure 2B4**) revealed that only exercised animals, but not control animals, discriminated between the novel and familiar object locations (*p* < 0.05, Student’s *t*-test), suggesting that control animals did not consolidate the information into long-term memory.

### NOVEL OBJECT RECOGNITION (NOR) MEMORY

We used the NOR task as a third assessment of the hypothesis that post-learning exercise can improve consolidation in aged dogs. This test paradigm consisted of a NOR acquisition trial, followed immediately by exercise (10 min) or rest (controls), and assessment of retention 1 and 24 h later.

NOR testing revealed that both exercise and control animals were are able to learn the task, but that an acute bout of post-acquisition exercise did not improve consolidation and performance either at short (1 h) or long-term (24 h) retention timepoints. Post-acquisition exercise appeared to even slightly impair performance at the 1 h timepoint. Specifically a repeated measure ANOVA revealed no interaction effect [*F*_(1,14)_ = 0.70, *p* = NS] or main effect of intervention [*F*_(1,14)_ = 0.14, *p* = NS, **Figure 3A1**] or object [*F*_(1,14)_ = 0.12, *p* = NS, **Figure 3A1**] in the acquisition phase of the task. A mixed factor ANOVA with treatment as the between subjects effect and object as the within subjects factor revealed no overall interaction effect [*F*_(1,14)_ = 0.31, *p* = NS], but an effect of object [*F*_(1,14)_ = 11.2, *p* < 0.05] but not treatment [*F*_(1,14)_ = 0.63, *p* = NS] at the 1 h timepoint. *Post hoc* testing revealed an increased exploration of the novel object only in control animals at the 1 h timepoint (*p* < 0.05, Bonferroni *post hoc* test, **Figure 3A2**). At the 24 h timepoint, analysis using a mixed factor ANOVA with treatment as the between subjects effect and object as the within subjects factor revealed no overall interaction effect [*F*_(1,13)_ = 0.00, *p* = NS], but an effect of object [*F*_(1,13)_ = 26.09, *p* < 0.01] but not treatment [*F*_(1,13)_ = 0.37, *p* = NS] and increased exploration of the novel object by both exercise and control animals at the 24 h timepoint (*p* < 0.01, Bonferroni *post hoc* test, **Figure 3B1**). There was no difference in the index of discrimination or DI between exercise and control groups at either the 1 h timepoint (*p* = NS; **Figures 3A3,A4**) or the 24 h timepoint (**Figures 3B2,B3**). These results reveal that both exercise and control animals are able to learn this task, and that an acute post-acquisition exercise bout does not improve consolidation or performance in the NOR task, contrasting with the benefits observed for OLM and the concurrent discrimination task.

### CHRONIC EXERCISE EFFECTS ON COGNITIVE PERFORMANCE IN AGED DOGS

In addition to testing how an acute bout of post-learning exercise affects cognitive performance, we investigated the effects of chronic daily exercise on cognitive function using two different chronic exercise approaches as specified beneath.

### OBJECT LOCATION MEMORY

In the first assessment of cognitive benefits of chronic exercise, animals in the exercise group underwent daily treadmill exercise (10 min/day) for 14 consecutive days, followed by a 48 h break from exercise, prior to cognitive testing. Exercise and control animals then underwent an OLM acquisition trial, and were tested for retention 1 and 24 h later.

OLM testing revealed significant benefits of chronic daily exercise on memory performance after 24 h of consolidation, but not at the 1 h timepoint. Specifically, a repeated measures ANOVA revealed no interaction [*F*_(1,12)_ = 0.01, *p* = NS] or main effect of exercise [*F*_(1,12)_ = 0.26, *p* = NS] or object location [*F*_(1,12)_ = 2.22, *p* = NS] during the acquisition phase (**Figure 4A1**). Similarly, there was no interaction effect [*F*_(1,13)_ = 0.6, *p* = NS] or main effect of exercise [*F*_(1,13)_ = 2.14, *p* = NS] or object location [*F*_(1,13)_ = 0.13, *p* = NS] at the 1 h timepoint (**Figure 4A2**). *t*-Test on the index of discrimination (*p* = NS; **Figure 4A2**) and the DI measure (*p* = NS, **Figure 4A3**) also revealed no effect of exercise.

At the 24 h timepoint, a repeated measures ANOVA showed a trend for interaction effect [*F*_(1,10)_ = 3.38, *p* = 0.09]. A significant treatment effect was also apparent 24 h post-acquisition [*F*_(1,10)_ = 12.16, *p* < 0.01, **Figure 4B1**], with *post hoc* analysis revealing that exercised animals, but not controls, showed significantly longer exploration of the object in the novel location than the object in the familiar location (*p* < 0.05, Bonferroni *post hoc* test, **Figure 4B1**). There was a significant effect of object location [*F*_(1,10)_ = 8.64, *p* < 0.05]. This was also confirmed by analysis of the index of discrimination (*p* < 0.05, **Figure 4B2**) but not by the DI measure (*p* = NS, **Figure 4B3**). These data suggest that chronic exercise boosts memory, particularly when the animal has had sufficient time to encode a memory.

### REVERSAL LEARNING TASK

Finally, we tested if aged dogs benefited from a chronic exercise intervention that occurred with learning. The task consisted of two phases. In the first phase (object discrimination learning), animals were required to discriminate between two objects and reach criterion performance (≥80% correct/session). Once animals learned the task to criterion, the reward contingencies were reversed, such that the previously negative stimulus became the positive stimulus and vice versa. This second phase (reversal) assesses cognitive flexibility, and is sensitive to frontal lobe function. For animals in the exercise group, each dog underwent daily exercise (10 min treadmill) immediately prior to the test session, beginning the day of preference testing and continuing through reversal learning. Days of exercise was determined by the number of days needed to reach criterion, which was on average 2–7 days for the acquisition phase and 7–14 days for the reversal phase.

The results revealed that daily exercise prior to the testing session facilitated reversal learning. Specifically, there was a significant interaction between exercise and trials [repeated measures ANOVA: *F*_(1,16)_ = 4.93, *p* < 0.05], with the exercise group making significantly fewer errors than controls in reversal learning (Bonferroni corrected *t*-test, *p* < 0.05, **Figure 5A1**). Overall, animals in both groups made significantly more errors in the reversal learning than in the initial acquisition learning [*F*_(1,16)_ = 41.47, *p* < 0.001], demonstrating that reversal learning is more difficult than the initial task acquisition phase. These results suggest that daily exercise concurrent with learning improves cognitive flexibility in aged dogs, allowing the animals to more rapidly learn that “the rules have changed.”

## DISCUSSION

This study was designed to evaluate the effects of exercise on cognitive function and memory consolidation. The results demonstrate that cognitive function can be improved by both short durations of increased physical activity as well as a program of daily exercise for 2 weeks. We found that exercise improved performance of aged beagles in several different behavioral tasks in a time-dependent manner. The improvements were robust at 24 h post-exercise; by contrast much smaller or insignificant effects were observed when animals were tested either immediately or 1 h after exercise (for summary see **Table 1**). Animals in the exercise group showed improved consolidation in the concurrent discrimination task and a spatial task. We also found improvements in a chronic exercise design in the object location and reversal learning tasks. It is important to note that we used aged canines (mean age of 10.6 year at the start of the study). This is potentially significant since human studies aiming to demonstrate the effect of exercise to improve memory in aging have resulted in variable outcomes (Madden et al., 1989; Colcombe and Kramer, 2003; Roig et al., 2013). Studies in rodents have shown that aged animals can learn and acquire novel information but impairment in retention is observed as the delay is increased (Driscoll et al., 2006; Bizon et al., 2009).

**Table 1:** The effect of exercise on different behavioral measures.

	Immediately post-exercise	1 h post-exercise	24 h post-exercise	48 h post exercise	48 h post exercise	72 h post-exercise	Learning	Reversal
Concurrent discrimination task	No significant difference between groups	NA	*p < 0.05	NA	NA	NA	NA
OLM task 1 h ITI	NA	No significant difference between groups	*p < 0.05	NA	NA	NA	NA
NOR task 1 h ITI	NA	No significant difference between groups	No significant difference between groups	NA	NA	NA	NA
OLM task after chronic exercise (14 days) with 1 h ITI	NA	NA	NA	No significant difference Between groups	NA	NA	NA
OLM task after chronic exercise (14 days) with 24 h ITI	NA	NA	NA	NA	*p < 0.05	NA	NA
Reversal learning task	NA	NA	NA	NA	NA	No significant difference between groups	*p < 0.05

OLM, object location memory; NOR, Novel object recognition; ITI, inter-trial interval; NA, not applicable, *p < 0.05 significant difference between exercise and control groups.

The concurrent discrimination tasks measures the ability to remember object–object association in the presence of active interference in the form of a list of objects (Buffalo et al., 1998; Broadbent et al., 2007). Aged primates have been reported to show deficits in performance on the concurrent discrimination task (Moss et al., 1981). However, this is the first study to use this task in canine subjects to show that acute exercise can help improve performance in this task. This task is different from the traditional two choice simple discrimination task, in that, the animals cannot solve the task based on the degree of familiarity with particular stimuli (recognition memory) because of the presence of interference (Gaffan and Murray, 1992). Our results also support this difference, in that while the aged control animals performed poorly in the concurrent discrimination task (pre-exercise versus post-exercise comparisons), they did not appear impaired in a NOR task. Although it has been suggested that the concurrent discrimination task in monkeys is based on familiarity, in humans it is shown to be MTL-dependent learning (Hood et al., 1999). It is important to note that while there was no difference in performance between the control and exercise groups immediately after the exercise, there was a significant improvement in performance 24 h the exercise session. This suggests that the effects observed in this study are because of longer term downstream effects of exercise rather than improved short-term memory.

Based on the results from the concurrent discrimination task where we observed post-exercise effects, we hypothesized that exercise improved memory consolidation rather than learning. To test this hypothesis animals were tested on an OLM task in a post-trial exercise paradigm. The OLM task is a measure of spatial discrimination and is considered to involve a hippocampus-dependent mechanism (Intlekofer et al., 2013). Similar to the concurrent discrimination task, we found that the effects of acute exercise on promoting memory function were only observed 24 h post-training in the OLM task. When animals were tested 1 h after the exercise sessions, they did not show any improved recollection of the spatial location of objects. This further supports the hypothesis that the effects of exercise that we observe in this study are not a result of arousal but longer term downstream affects that are either recruited, or persist for as long as 24 h after a single bout of exercise. It is also important to note that in this study exercise training occurred after the acquisition trial. We have recently demonstrated that post-trial exercise intervention facilitates memory consolidation in amnestic mild cognitive impairment (aMCI) patients (Segal et al., 2012). It should be noted however, that the Segal study, involved recall of emotional images. The task used by Segal and colleagues is thus different from concurrent discrimination task and OLM tasks. Moreover, testing was done at 1 h post-exercise which is distinct from the current data which shows improved performance 24 h post-treatment. However, it is evident that both human and canine data support the role of exercise as a mechanism to improve memory consolidation. Our findings confirm and extend human studies on post-trial learning mechanisms and attest to the value of canine as a model of aging.

The aged beagles were also tested in a NOR task designed to evaluate the effects of exercise on a task that utilized minimal spatial learning and hippocampal involvement. Interestingly there was no significant difference in the discrimination ability of animals in exercise and control groups in this task. Performance in the NOR task is thought to rely primarily on the perirhinal cortex (Aggleton et al., 1997; Ennaceur and Aggleton, 1997; Aggleton and Brown, 2005). In this study we found that aged beagles in the control groups were able to distinguish novel from familiar, this effect persisted in the exercise group also. Thus it may be possible that the perirhinal cortex is relatively spared in aging. These data are similar to those in aged rodents where long-term exercise improves recall for OLM but not object recognition memory (Siette et al., 2013). However, it is important to note that there may be alternate explanations which could explain the lack of effect of exercise in the NOR task. It is possible that there was no difference between the two groups because of ceiling effects in the control animals rather than a lack of efficacy on perirhinal-dependent function. Further studies will be needed to determine the more plausible explanation.

A novel finding that emerges from this study is the idea that acute exercise impacts the MTL memory systems in a time-dependent manner. It is important to compare the effects of acute exercise vs. chronic exercise on improving cognitive function in the canine model using similar tasks. Therefore in a chronic exercise study, dogs were allowed to run on the treadmill for 14 days (10 min/day) and were then given a break of 48 h before being tested on the object location task. Our results indicated that exercise had a lasting a benefit that resulted in improved OLM even 48–72 h after the last exercise session. This is consistent with several other studies which have shown lasting improvements in cognitive function following increased physical activity. It is important to note that no effect of exercise was observed 1 h post-acquisition even after a chronic exercise regime. This agrees with data from acute exercise treatment and suggests that exercise-induced improvement in learning and memory involves consolidation and retrieval rather than memory acquisition. In a recent article Roig et al., suggested an interesting dissociation between the effects of acute and chronic exercise on human memory (Segal et al., 2012; Roig et al., 2013). The authors suggested that while acute exercise impact memory consolidation in a time-dependent manner, chronic exercise is more likely to induce physiological adaptations which prime the molecular machinery of the brain to augment memory processing. This is consistent with our findings that 24 h retention of OLM was improved even when testing was conducted 48–72 h after the last exercise session. Overall, our findings show that acute and chronic exercise treatment regimes share the ability to facilitate memory consolidation and are effective in improving cognitive function.

Finally the animals were tested on a reversal learning test, which is used to measures executive function. The NIH toolbox cognitive function battery defines executive function as control of goal directed behavior (Snigdha et al., 2013; Weintraub et al., 2013). It includes concept formation and manipulation of information in response to environmental cues and requires active involvement of the frontal lobe. Typically the reversal learning task which requires learning and then unlearning a rule based on change in reinforcement contingencies is a useful measure of executive function (Tapp et al., 2003, 2004). In this study we found that exercise did not affect performance on the learning phase but significantly improved the errors to criterion in the reversal phase of the reversal learning task. This suggests a selective improvement in the reversal but not learning phase of the task, with the animals in the control group making more perseverative responses. Our data suggest that exercise may not impact simple cognitive functions as required in the first (learning) phase of the task. However, discrimination reversals which require subjects to show inhibitory control and discontinue prepotent responses to a previously correct stimulus, is improved by exercise.

We would like to point out that while aged beagles provide a unique advantage as a model for studies of aging, there are some limitations of using this model. For instance, due to limited availability of aging dogs all tests reported here were conducted in the same animals and the low number of animals for single trial tests may reduce power to detect some effects. Furthermore, like humans, aged dogs demonstrate domain-specific deficits in learning and memory as well as individual variability (Adams et al., 2000; Tapp et al., 2003). It is important to note that individual variability can have a confounding effect in studies (as seen in this study). We have, however, tried to control for some of these effects in this study by distributing animals from all three dietary groups equally between control and exercise groups as stated previously. We would also like to note that although the handling of the exercise group and not controls during the exercise training may lead to some confounds, it would stand to reason that any effects of handling would be expected to have the greatest effects early in testing, where no treatment effects were observed.

Finally, although age-related effects were not established in this paper, we have in the past demonstrated age-related differences in performance in many of the tasks used here (Tapp et al., 2003; Cotman and Head, 2008). Thus, despite potential confounds, our data clearly suggest that the mechanisms to improve overall memory and cognitive function remain accessible in aged animals and can be re-engaged with physical exercise.

In summary, the results of this study indicate that acute and chronic exercise are both effective against the functional deficits associated with cognitive aging. Exercise may therefore be a viable training mechanism to improve cognitive reserve in the brain and bolster resiliency against the consequences of brain aging.

## Conflict of Interest Statement

The authors declare that the research was conducted in the absence of any commercial or financial relationships that could be construed as a potential conflict of interest.
